# Thrombolysis increases the risk of persistent headache attributed to ischemic stroke: A prospective observational study

**DOI:** 10.1002/brb3.3447

**Published:** 2024-03-07

**Authors:** Yi Zhang, Wensheng Qu, Cenk Ayata, Qianqian Kong, Jing Zhao, Xirui Zhou, Dan He, Zhiyuan Yu, Hao Huang, Xiang Luo

**Affiliations:** ^1^ Department of Neurology, Tongji Hospital, Tongji Medical College Huazhong University of Science and Technology Wuhan Hubei China; ^2^ Hubei Key Laboratory of Neural Injury and Functional Reconstruction Huazhong University of Science and Technology Wuhan China; ^3^ Department of Radiology, Massachusetts General Hospital Harvard Medical School Charlestown Massachusetts USA; ^4^ Department of Neurology, Massachusetts General Hospital Harvard Medical School Boston Massachusetts USA; ^5^ Department of Neurology, National Key Clinical Department and Key Discipline of Neurology, The First Affiliated Hospital Sun Yat‐sen University Guangzhou China

**Keywords:** intravenous thrombolysis, ischemia, persistent headache attributed to past ischemic stroke

## Abstract

**Background and objective:**

Persistent headache attributed to ischemic stroke (PHPIS) is increasingly acknowledged and was added to the 2018 ICHD‐3. Intravenous thrombolysis (IVT) is a common treatment for acute ischemic stroke. It remains unknown whether this treatment influences the occurrence of a persistent poststroke headache. We aimed to describe the incidence and clinical characteristics of persistent headaches occurring after acute ischemic stroke in patients with or without IVT and explore the risk factors.

**Methods:**

A prospective observational study was performed between the 234 individuals who received IVT and 226 individuals without IVT in 5 stroke units from Wuhan, China. Subjects were followed for 6 months after stroke via a structured questionnaire.

**Results:**

Age, gender, vascular risk factors, and infarct location/ circulation distribution did not differ between the groups, although IVT group had higher initial NIHSS scores. At the end of the follow‐up, 12.0% (55/460) of subjects reported persistent headaches after ischemic stroke. The prevalence of persistent headache was significantly higher in the IVT group than non‐IVT group (15.4% vs. 8.4%, *p* = .021). Patients with younger age (*p *= .033; OR 0.97; 95% CI 0.939–0.997), female sex (*p *= .007; OR 2.40; 95% CI 1.269–4.520), posterior circulation infarct (*p *= .024; OR 2.19; 95% CI 1.110–4.311), and IVT (*p *= .005; OR 2.51; 95% CI 1.313–4.782) were more likely to develop persistent headache after ischemic stroke.

**Conclusion:**

The potential influence of IVT should be considered when assessing persistent poststroke headache. Future studies will investigate the underlying mechanisms.

## Abstract 2

This prospective observational study aimed to investigate the characteristics of persistent headache attributed to past ischemic strok (PHPIS) and the impact of intravenous thrombolysis on this headache in patients with acute ischemic stroke. The study included 460 patients with acute ischemic stroke and found that intravenous thrombolytic therapy was an important risk factor for the presence of PHPIS.

## 1 INTRODUCTION

Stroke is the leading cause of death and disability worldwide (Wang et al., 2017). Headache is a common symptom or even a complicating disease secondary to the stroke attack (Harriott et al., 2020). Both stroke and headache cause neurological disability, and the occurrence of poststroke headache is associated with a lower quality of life (Tabeeva, 2021; Westerlind et al., 2020). Poststroke headache is a type of headache attributed to a stroke, which typically develops in temporal relation to the onset of the stroke and significantly worsens or improves in parallel with the worsening or improvement of the stroke.

In the literature, 33.5% of stroke patients experienced a headache at stroke onset, 7–23.0% had persistent headaches for 3 months, and early emergence of headaches predicted persistent headaches at 6 months (Lai et al., 2018). The persistent headaches still exist although the stroke is improved. In the 2018 International Classification of Headache Disorders, 3rd edition (ICHD‐3), this has been termed as “persistent headache attributed to past ischemic stroke” (PHPIS), which is a subset of headaches that begins at stroke onset and continues months to years thereafter (Olesen et al., 2018). As a new type of headache, the knowledge of PHHIS is still poor.

Intravenous thrombolysis (IVT) is a major therapeutic modality in acute ischemic stroke (Powers et al., 2019). IVT can restore blood supply, reduce neural injury, significantly relieve symptoms at the acute stage, and improve long‐term prognosis (Goyal et al., 2015; Todo et al., 2019). However, it also increases the risk of intracerebral hemorrhage and ischemia‐reperfusion injury (Demaerschalk, 2007; Ferro et al., 2021; Ng et al., 2021) may be through promoting inflammation (Dodick, 2018; Harriott et al., 2020), vascular endothelial damage (Leira et al., 2002) and free radicals (Oztanir et al., 2014; Sladojevic et al., 2014). Therefore, the IVT was supposed to predispose the appearance of PHPIS and act as a risk factor. Here, we performed an observational study in objects with IVT or not, to examined the prevalence, clinical characteristics, and risk factors of PHPIS, and find the correlations between IVT and PHPIS.

## 2 METHODS

### 2.1 Participants

A prospective observational study was performed on inpatients from 5 stroke units in Wuhan, China, during the period spanning October 2020 to January 2022. Clinical data were collected from eligible individuals if they were (1) ≥18 years old and (2) diagnosed with acute ischemic stroke (within 12 h after onset) according to the criteria of ICD‐10 Code I63.9 (cerebral infarction). Patients were excluded if they (1) had de novo headache better accounted by another ICHD‐3 diagnosis except cerebral infarction (Code 6.1.1), such as concomitant intracranial hemorrhage, cranial venous sinus thrombosis, and cervical or intracranial artery dissection; (2) received endovascular treatments like angioplasty, stenting, or intraarterial thrombectomy; (3) took drugs that may influence the presence of headaches, such as nitroglycerin, nicardipine, dipyridamole, and cilostazol (Ashina et al., 2021; Young, 2004); (4) had communication problems because of aphasia, severe dysarthria, or dementia; (5) had neuropsychological disorders like severe depression, anxiety, and schizophrenia; (6) had headache confirmed as recurrence of a previous headache before ischemia (previous headache was defined by interview according to ICHD‐3); and (7) declined follow‐up.

This study was approved by the Ethics Committee of Tongji Hospital (No. TJ‐IRB20210107) and conducted according to the Declaration of Helsinki. According to ethics guidelines, the requirement of written informed consent was waived because anonymization was used in this observational study and there was no risk of harm to the patients.

**FIGURE 1:**
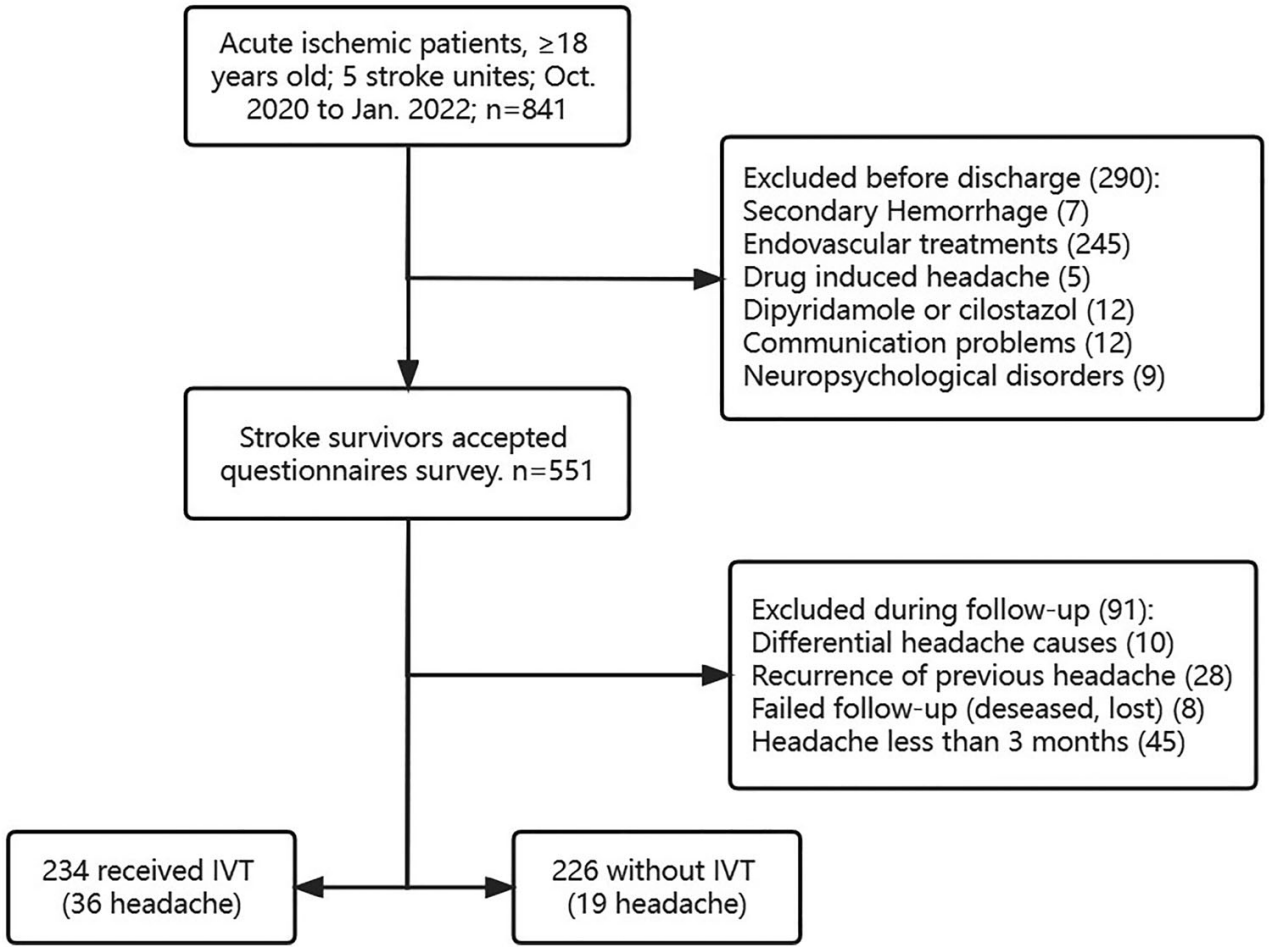
Flow chart of patient selection.

According to the Chinese Stroke Association guidelines (Liu et al., 2020), which is similar to the guidelines from the American Heart Association/American Stroke Association (Powers et al., 2019), acute ischemic stroke patients received IVT using recombinant tissue plasminogen activator (rt‐PA, alteplase, 0.9 mg/kg, maximum dose 90 mg, over 60 min with initial 10% bolus over 1 min). These patients were enrolled in the IVT group, while others were included in the non‐IVT control group (Figure 1).

### 2.2 Clinical assessments

The following data were collected: demographics; vascular risk factors including hypertension (previously documented or undergoing antihypertensive therapy), diabetes (previously documented or prescribed insulin or an oral antiglycemic), hyperlipidemia (LDLc ≥ 2.6 mmol/L or prescribed lipid‐lowering drugs), smoking, and alcohol consumption; history of atrial fibrillation or stroke; brain imaging (CT/MR); initial NIH stroke scale (NIHSS) within the first 12 h after ischemia onset and Alberta Stroke Program Early CT Score (ASPECTS, a semiquantitative grading system that measures the extent of early ischemic changes) (Yoo et al., 2016). Brain CT and/or MRI on admission were used to classify the distribution of infarcts according to anatomical and vascular distribution (anterior circulation: middle and anterior cerebral artery, posterior circulation: posterior cerebral artery, or both).

### 2.3 Headache assessments

With face‐to‐face interviews by a trained neurologist who has been blinded, characteristics of headaches were recorded on the admission and in the third and sixth month postischemia, followed by online interviews later. A standardized questionnaire (Table S1) was prepared as previously reported (Olesen et al., 2000), which records the cause, characteristics (location, intensity, headache type, concomitant symptoms, and daily influence) (Olesen et al., 2018), and headache treatments used before stroke, during acute stroke, and after stabilization (dominant symptoms stop getting worse). Standard evaluations including the Visual Analogue Scale (VAS) and Headache Impact Test‐6 (HIT‐6) were performed to determine the severity and impact of poststroke headache on daily life (Aicher et al., 2012; Haywood et al., 2021).

According to the 2018 ICHD‐3 Code 6.1.1.2, PHPIS is defined as the development of headache simultaneously with or in close temporal relationship to signs or other evidence of ischemic stroke confirmed by imaging and clinical symptoms, the persistence of symptoms for more than 3 months, and the lack of another ICHD‐3 diagnosis that would better account for the occurrence of persistent headache. The diagnosis of PHPIS was given after 6 months of follow‐up. Headache was described as migraine‐like (referring to Code 1.5), tension‐type headache‐like (referring to Code 2.4), or others (Olesen et al., 2018).

The characteristics of headaches were described as follows. The appearance of the first attack was grouped according to the initial time after ischemia, within 1 h, 1–24 h, or 24 h later. The location of the headache was categorized as left, right, or bilateral. The quality of headache was categorized as pulsating, tension‐type, throbbing, or other. Accompanying phenomena like nausea, photophobia, phonophobia, and dizziness were recorded. The frequency of headache was classified as less than 7, 7–14, or more than 14 headache days per month. The intensity of headache was graded as mild (VAS ≤ 3), moderate (VAS = 4–6), or severe (VAS ≥ 7) pain (Collins et al., 1997). Depending on the HIT‐6 scale, daily life impact was estimated as ≤ 50, no/little impact; 50–55, some impact; 56–59, substantial impact; and ≥ 60, severe impact (Haywood et al., 2021).

Medications were monitored to account for possible medication overuse headache (ICHD‐3 Code 8.2) (Olesen et al., 2018). Typical treatments for ischemia and its comorbidities were given according to the guidelines from the Chinese Medical Association and National Institutes of Health (USA). Medications (like dipyridamole and cilostazol) and paregorics that might influence the headache were recorded (Westergaard et al., 2014). Although neuropsychological disorders are not quantified by scales, possible events were recorded to estimate the influence on the headache.

### 2.4 Statistical analysis

The sample size was based on the available data and no statistical power calculation was done upfront. This was the primary analysis of our collected data. Statistical analyses were performed using the IBM SPSS statistical package (version 25.0, IBM Corp., Armonk, NY, USA). Continuous variables consistent with normal distribution were compared via independent‐sample *t*‐test, and results were reported as mean ± standard deviation (SD). Continuous variables without normal distribution were compared via the Mann–Whitney *U* test, and results were represented by median and interquartile range. Categorical variables were analyzed by chi‐square test or Fisher's test, and results were reported as numbers and percentages. Baseline variables that were considered clinically relevant or that showed univariable relationships with the dependent variables were entered into multiple regression. Binary logistic regression analysis was performed using backward elimination to evaluate possible risk factors for headache. An internal validation using a bootstrap resampling process was conducted to evaluate the performance of this model. Multicollinearity was confirmed to be absent by a variance inflation factor of less than 10 across all variables. Odds ratios (ORs) and 95% confidence intervals (CIs) of variables were determined. *p *< .05 was considered to indicate statistical significance.

## 3 RESULTS

### 3.1 Participants

During the in‐hospital period, 290 patients were excluded for reasons shown in Figure 1, while 551 participants received the questionnaire interview. In total, headache was observed in 25.2% (139/551) patients after ischemia. Among these patients, headaches ceased in 32.4% (45/139) of cases within 3 months poststroke, 33.7% (28/83) cases with persistent headache reported recurrence as previously existed; and, 10 cases were attributed to hemorrhage (1), trauma (2), cervical disease (5), and somatization disorder (2). As a result, a total of 55 subjects were diagnosed as PHPIS.

The demographics and clinical characteristics of the two groups are presented in Table 1. When comparing between IVT and non‐IVT groups, no statistical differences were found in age, gender, medical history (hypertension, hyperlipemia, diabetes, atrial fibrillation, previous stroke, smoking, and alcohol intake), infarct location, and vascular distribution. The IVT group had higher initial NIHSS and ASPECT scores compared with that in non‐IVT group (*p* < .05).

**TABLE 1:** Demographics and clinical characteristics.

	Non‐IVT (*n *= 226)	IVT (*n* = 234)	*p* Value
Age (years)	60.0 (54.0−69.0)	64 (56−70)	.090
Males, *n* (%)	164 (72.6)	164 (70.1)	.556
Hypertension, *n* (%)	147 (65.0)	170 (72.6)	.078
Diabetes, *n* (%)	70 (31.0)	63 (26.9)	.338
Hyperlipemia, *n* (%)	24 (10.6)	28 (12.0)	.648
Atrial fibrillation, *n* (%)	8 (3.5)	17 (7.3)	.078
Previous stroke, *n* (%)	37 (16.4)	37 (15.8)	.870
Smoking, *n* (%)	87 (38.5)	111 (47.4)	.053
Alcohol intake, *n* (%)	72 (31.9)	80 (34.2)	.595
Infarct location, *n* (%)			.481
Left	104 (46.0)	96 (41.0)	
Right	81 (35.8)	96 (41.0)	
Bilateral	41 (18.1)	42 (17.9)	
Circulation, *n* (%)			.500
Anterior	144 (63.7)	140 (59.8)	
Posterior	43 (19.0)	55 (23.5)	
Both	39 (17.3)	39 (16.7)	
ASPECT	9 (8–9)	9 (8–10)	.010^a^
Initial NIHSS	4 (3−6)	7 (5−9)	< .001^a^

Abbreviations: ASPECT, Alberta Stroke Program Early CT Score; IVT, intravenous thrombolysis; NIHSS, National Institute of Health stroke Scale.

^a^ *p *< .05 vs. non‐IVT group.

### 3.2 Characteristics of PHPIS

Relative to that in the non‐IVT group, the prevalence of PHPIS was significantly higher in the IVT group (15.4%, 36/234, vs. non‐IVT group, 8.4%, 19/226; Table 2). No statistically significant difference was observed between the two groups regarding headache severity (VAS score), frequency, location, quality, or influence on daily life (HIT‐6 score). In the IVT group, persistent headaches were more likely to be initiated during the first day after stroke onset in IVT (38.9%) than non‐IVT group (10.5%). Headaches in the IVT group were more likely to be characterized as moderate‐severe intensity, bilateral, and pulsating/stabbing quality; dizziness was a conspicuous accompanying symptom in 55.6% of the headache cases after IVT.

**TABLE 2:** Characteristics of PHPIS.

	Non‐IVT (*n* = 226)	IVT (*n* = 234)	*p* Value
Persistent headache, *n* (%)	19 (8.4)	36 (15.4)	.021^a^
First attack, *n* (%)			.036^a^
Within 1 h after stroke onset	9 (47.4)	16 (44.4)	
1–24 h after stroke onset	2 (10.5)	14 (38.9)	
24 h later after stroke onset	8 (42.1)	6 (16.7)	
Severity (VAS score), *n* (%)			.465
Mild (≤ 3)	17 (89.5)	28 (77.8)	
Moderate‐severe (≥ 4)	2 (10.5)	8 (22.2)	
Frequency			.539
>14 days per month	3 (15.8)	10 (27.8)	
7–14 days per month	5 (26.3)	10 (27.8)	
< 7 days per month	11 (57.9)	16 (44.4)	
Location, *n* (%)			.186
Left	6 (31.6)	10 (27.8)	
Right	10 (52.6)	12 (33.3)	
Bilateral	3 (15.8)	14 (38.9)	
Quality, *n* (%)			.453
Pulsating	8 (42.1)	17 (47.2)	
Tension‐type	10 (52.6)	13 (36.1)	
Stabbing	0 (0)	3 (8.3)	
Others	1 (5.3)	3 (8.3)	
Duration			.320
Less than 24 h	15 (78.9)	32 (88.9)	
More than one day	4 (21.1)	4 (11.1)	
Accompanying symptoms			
Dizziness	5 (27.8)	20 (55.6)	.054
Nausea	1 (5.6)	4 (11.1)	.507
HIT‐6, *n* (%)			.707
No/little (≤50)	9 (47.4)	19 (52.8)	
Some (51−55)	5 (26.3)	9 (25.0)	
Substantial (56−60)	4 (21.0)	4 (11.1)	
Severe (> 60)	1 (5.3)	4 (11.1)	

Abbreviations: HIT‐6, Headache Impact Test‐6; IVT, intravenous thrombolysis; PHPIS, persistent headache attributed to past ischemic stroke; VAS, Visual Analogue Scale.

^a^ *p *< .05 vs. non‐IVT group.

Persistent headache cases were more likely to be tension‐type‐like (49.0%) than migraine‐like (38.2%). Most headache attacks (85.5%, 47/55) lasted less than 24 h and had limited influence on daily life (Table 2). Only 6 (10.9%) cases took analgesics during some attacks and no medication overuse headache was documented.

### 3.3 Risk factors for PHPIS

Traditional vascular risk factors such as hypertension, hyperlipemia, diabetes, atrial fibrillation, previous stroke, smoking, and alcohol intake did not differ between subjects who developed PHPIS and those who did not. The initial NIHSS and ASPECTS also did not differ. In contrast, females, subjects who received IVT, and posterior circulation involvement had higher risk of PHPIS compared with the nonheadache cohort (Table 3). Multiple regression with stepwise backward elimination confirmed younger age, female sex, IVT (OR 2.51; 95% CI 1.313–4.782), and posterior circulation involvement as independent risk factors (Table 4, see Table S2 for details).

**TABLE 3:** Risk factors for PHPIS.

	Nonheadache (*n* = 405)	Headache (*n* = 55)	*p* Value
Age (year)	63 (55–70)	60 (52–66)	.069
Males, *n* (%)	295 (72.8)	33 (60.0)	.048^a^
Hypertension, *n* (%)	283 (69.9)	34 (61.8)	.226
Diabetes, *n* (%)	117 (28.9)	16 (29.1)	.975
Hyperlipemia, *n* (%)	57 (14.1)	6 (10.9)	.522
Atrial fibrillation, *n* (%)	23 (5.7)	2 (3.6)	.531
Previous stroke, *n* (%)	62 (15.3)	12 (21.8)	.218
Smoking, *n* (%)	174 (43.0)	24 (43.6)	.925
Alcohol intake, *n* (%)	136 (33.6)	16 (29.1)	.507
IVT, *n* (%)	198 (48.9)	36 (65.5)	.021^a^
Circulation, *n* (%)			.009^a^
Anterior	260 (64.2)	24 (43.6)	
Posterior	79 (19.5)	19 (34.5)	
Both	66 (16.3)	12 (21.8)	
NIHSS	6 (3–8)	6 (3–8)	.689
ASPECT	9 (8–9)	9 (8–9)	.827

Abbreviations: ASPECT, Alberta Stroke Program Early CT Score; IVT, intravenous thrombolysis; NIHSS, National Institute of Health stroke Scale; PHPIS, persistent headache attributed to past ischemic stroke.

^a^ *p *< .05 vs. nonheadache group.

**TABLE 4:** Stepwise multiple regression analysis for risk factors.

Factors	OR (95% CI)	*p* Value
Age	0.97 (0.939–0.997)	.033^a^
Female sex	2.40 (1.269–4.520)	.007^a^
IVT	2.51 (1.313–4.782)	.005^a^
Circulation		
Anterior	Ref.	
Posterior	2.19 (1.110–4.311)	.024^a^
Both	2.09 (0.962–4.519)	.063

Abbreviations: CI, confidence interval; IVT, intravenous thrombolysis; OR, odds ratio.

Binary logistic regression, backward, adjusted analysis includes observed categorical variable (sex, hypertension, hyperlipemia, diabetes, atrial fibrillation, previous stroke, smoking, alcohol intake, IVT, and circulation) and continuous variables (age, NIHSS, and ASPECT scores).

^a^ *p *< .05.

## 4 DISCUSSION

Headache is a common but under‐appreciated symptom in ischemic stroke patients. A 10–12% prevalence of persistent headache after stroke has been detected in observational studies spanning 16 months (Jonsson et al., 2006) to 3 years after stroke (Hansen et al., 2015), although the prevalence appeared to decrease over time (Naess et al., 2010; Osama et al., 2018). In this study, headache was observed in 25.2% of patients after ischemia, and 12.0% were diagnosed with PHPIS after 3 months of follow‐up in the present study.

The characteristics of headaches following an ischemic stroke usually vary. In our study, patients mostly experienced mild headaches lasting no more than 24 h, had migraine or tension‐type‐like headaches, and rarely reported substantial impact on daily life. This is consistent with previous reports, where 50.0% of headaches were tension‐type‐like (Hansen et al., 2015), and usually mild (Carvalho Dias et al., 2020), although moderate to severe pain was also experienced (Jonsson et al., 2006). Accompanying symptoms like nausea/vomiting, phonophobia, and photophobia were reported (Hansen et al., 2015). Interestingly, dizziness was a frequent symptom accompanying headaches especially in IVT patients in the present study. Although medication overuse was previously reported in 6.25% cases with persistent headache following stroke (Hansen et al., 2015), and even 30% of cases with persistent headache after first‐ever ischemic stroke (Lebedeva et al., 2022), analgesic drugs were rarely taken by patients in this Chinese cohort. Patients with mild or moderate headaches usually did not take analgesics. The difference in population and culture should also be considered since the prevalence of medication overuse headache is intrinsically lower in China (Dong et al., 2015).

We also found that posterior circulation was associated with the PHPIS. Individuals with vertebrobasilar stroke were noted to have a higher probability of stroke‐related headache (Seifert et al., 2018). These findings suggest that infarct distribution is an important determinant for stroke‐associated headache. The reasons for this are not clear but may be related to perivascular innervation in posterior locations such as the trigeminal nucleus and thalamus. In another study using lesion mapping in the acute phase of stroke, individuals with lesions located in the insular cortex/cerebellum and somatosensory cortex were likely to have poststroke headache (Osama et al., 2018; Seifert et al., 2018). Ischemia might lead to dysfunction of the trigeminal nerves, serotonergic nuclei in the brainstem, somatosensory cortex, and even pain‐sensitive structures like dura mater, leading to headache (Kim et al., 2008; Mitsias et al., 2006). Furthermore, following ischemia, persistent chronic headaches may be linked to central sensitization of pain pathways (Woolf, 2011).

We also found a strong association between IVT and PHPIS. More PHPIS developed after IVT. Coincidently, another recanalization therapy, endovascular thrombectomy potentiated the de novo headache in ischemic stroke (Gallo et al., 2022). The mechanism has not been determined, but possibly due to vascular endothelial damage (Leira et al., 2002), ischemia‐reperfusion injury, free radicals and cytokines (Oztanir et al., 2014; Sladojevic et al., 2014), increased vascular permeability, and peripheral nerves stimulation (Ansar et al., 2014; Gipponi et al., 2010; Leira et al., 2002). Considering its short half‐life, rt‐PA is unlikely to be the direct trigger for persistent headache but could facilitate secondary injury, such as direct excitotoxicity, impairment of gamma‐aminobutyric acid inhibitory interneurons, blood‐brain barrier disruption, and excessive oxygen‐free radicals (Kidwell et al., 2008; Parathath & Tsirka, 2006; Yepes et al., 2009).

There are several limitations to the present study. First, without a randomized controlled design, the influence of IVT on PHPIS could not be directly tested by fully evaluating confounders. Second, although a multiple regression analysis accounting for age, sex, hypertension, hyperlipemia, diabetes, atrial fibrillation, previous stroke, smoking, alcohol intake, NIHSS and ASPECT, IVT, and arterial territory was carried out, new protocols are needed to evaluate these factors and other potential confounders (e.g., lesion size and detailed headache history). Third, bias may have resulted from limited cohort size and exclusions. Fourth, regional and ethnic differences were not observed in the present study.

## 5 CONCLUSIONS

We found that IVT was an important risk factor for the presence of PHPIS. Although the pain is usually mild‐to‐moderate and has limited influence on daily life, more attention must be paid to this newly recognized headache. Future studies will explore possible mechanisms utilizing, for example, transcranial Doppler ultrasound and functional MRI and identify prevention and treatment options for of IVT‐related persistent headaches.

## AUTHOR CONTRIBUTIONS

**Yi Zhang**: Conceptualization; investigation; writing—original draft; methodology; writing—review and editing; formal analysis; data curation; validation; software. **Wensheng Qu**: Conceptualization; methodology; investigation; validation; formal analysis; supervision; writing—original draft; writing—review and editing; data curation. **Cenk Ayata**: Project administration; supervision. **Qianqian Kong**: Data curation. **Jing Zhao**: Data curation. **Xirui Zhou**: Data curation. **Dan**: Data curation. **Zhiyuan Yu**: Supervision; funding acquisition. **Hao Huang**: Supervision. **Xiang Luo**: Supervision; funding acquisition; project administration; resources.

## CONFLICT OF INTEREST STATEMENT

The authors declared no potential conflicts of interest with respect to the research, authorship, and/or publication of this article.

### PEER REVIEW

The peer review history for this article is available at https://publons.com/publon/10.1002/brb3.3447.

## Supporting information

Table S1 Headache questionnaires.
Table S2 Univariate and stepwise multiple regression analysis of risk factors for PHPIS.

## Data Availability

Data could be achieved upon reasonable request to the corresponding author.
