# Supplementary material for: Thrombolysis increases the risk of persistent headache attributed to ischemic stroke: A prospective observational study
Source: Brain Behav. 2024 Mar 7;14(3):e3447. doi: 10.1002/brb3.3447 (PMC10918606; doi:10.1002/brb3.3447)
Supplement: Supplementary file 1 — Table S1 Headache questionnaires. Table S2 Univariate and stepwise multiple regression analysis of risk factors for PHPIS. [file BRB3-14-e3447-s001.docx]

**TableS1 Headache questionnaires**

| **Questions** | | **Yes** | **No** |
| --- | --- | --- | --- |
| 1. **Allergy** | | 1 | 0 |
| Specify: | | | |
| 1. **Inherited disorders** | | **Yes** | **No** |
| 1. Heart disease | | 1 | 0 |
| 1. Headache | | 1 | 0 |
| 1. Seizures | |  |  |
| Other: | | | |
| 1. **Family history** | | **Yes** | **No** |
| 1. Headache | | 1 | 0 |
| 1. Heart disease | | 1 | 0 |
| 1. Strokes | | 1 | 0 |
| 1. Seizures | | 1 | 0 |
| Other: | | | |
| 1. **Other disorders at present or before** | | **Yes** | **No** |
| 1. Head trauma   □ within 3m □ 3-6m before □6-12m before □ 1y before | | 1 | 0 |
| 1. Cerebral hemorrhage   □ within 3m □ 3-6m before □6-12m before □ 1y before | | 1 | 0 |
| 1. Ischemic stroke/ TIA   □ within 3m □ 3-6m before □6-12m before □ 1y before | | 1 | 0 |
| 1. Encephalitis/meningoencephalitis   □ within 3m □ 3-6m before □6-12m before □ 1y before | | 1 | 0 |
| 1. Cranial venous sinus thrombosis   □ within 3m □ 3-6m before □6-12m before □ 1y before | | 1 | 0 |
| 1. Carotid or intracranial artery dissection   □ within 3m □ 3-6m before □6-12m before □ 1y before | | 1 | 0 |
| 1. Unruptured aneurysm   □ within 3m □ 3-6m before □6-12m before □ 1y before | | 1 | 0 |
| 1. Intracranial tumor   □ within 3m □ 3-6m before □6-12m before □ 1y before | | 1 | 0 |
| 1. Epilepsy   □ within 3m □ 3-6m before □6-12m before □ 1y before | | 1 | 0 |
| Other:  □ within 3m □ 3-6m before □6-12m before □ 1y before | | | |
| 1. **Medicines taken in the last 3 months** | | **Yes** | **No** |
| 1. Triptans | | 1 | 0 |
| 1. NSAIDS | | 1 | 0 |
| 1. Flupentixol melitracen | | 1 | 0 |
| 1. Muscle relaxant | | 1 | 0 |
| Other: | | | |
| Specify: (types, usage and dosage)  1.  2.  3.  4. | | | |
| 1. **Previous headache in the last 6 months** | | **Yes** | **No** |
| 1. Previous headache | | 1 | 0 |
| If no, going to question 7 | | | |
| 1. Type Diagnosed: | | | |
| Migraine | | 1 | 0 |
| Tension-type headache | | 1 | 0 |
| Neuralgia | | 1 | 0 |
| Drug dependence headache | | 1 | 0 |
| Other: | | | |
| 1. Aura symptoms | | | |
| Group A | Blurred vision | 1 | 0 |
|  | Dark spots/floaters | 1 | 0 |
|  | Bright light/glare | 1 | 0 |
|  | Distorted vision | 1 | 0 |
|  | Other: | | |
| Group B | Nausea | 1 | 0 |
|  | Vomiting | 1 | 0 |
|  | Light sensitivity | 1 | 0 |
|  | Noise sensitivity | 1 | 0 |
|  | Numbness | 1 | 0 |
|  | Weakness | 1 | 0 |
|  | dizziness | 1 | 0 |
|  | Fever | 1 | 0 |
|  | Others: | | |
| Group C | Difficulty speaking | 1 | 0 |
|  | Enunciation unclear | 1 | 0 |
|  | Other: | | |
| Group D | Limb weakness | 1 | 0 |
|  | Ataxia | 1 | 0 |
|  | Other: | | |
| Other: | | | |
| 1. Duration | | | |
| ≤30 minutes | | 1 | 0 |
| 30 min- 4 h | | 1 | 0 |
| 4 h- 24h | | 1 | 0 |
| >1 day | | 1 | 0 |
| 1. Frequency | | | |
| ≤2 days every month | | 1 | 0 |
| 2-7 days every month | | 1 | 0 |
| 7-14 days every month | | 1 | 0 |
| >15 days every month | | 1 | 0 |
| 1. headaches occur on any particular day of the week or time of day | | 1 | 0 |
| Specify: | | | |
| 1. Severity (0-10, “0” means no pain, “10” means unbearable pain) | | | |
| Mild (0-3) | | 1 | 0 |
| Moderate (4-6) | | 1 | 0 |
| Severe (7-10) | | 1 | 0 |
| 1. Headache characteristics | | | |
| Pulsating | | 1 | 0 |
| Throbbing | | 1 | 0 |
| Stabbing | | 1 | 0 |
| Other: | | | |
| 1. Headache location: | | | |
| Left side | | 1 | 0 |
| Right side | | 1 | 0 |
| Both | | 1 | 0 |
| 1. Aggravation by: | | | |
| Exercise | | 1 | 0 |
| Stress | | 1 | 0 |
| Change in weather | | 1 | 0 |
| Alcohol | | 1 | 0 |
| Smoke | | 1 | 0 |
| Noise | | 1 | 0 |
| Dizziness | | 1 | 0 |
| Other: | | | |
| 1. Medicines taken for headache | | | |
| Triptans | | 1 | 0 |
| NSAIDS | | 1 | 0 |
| Opioid | | 1 | 0 |
| Ergots | | 1 | 0 |
| Other: | | | |
| Specify: (names, usage and dosage)  1.  2.  3.  4. | | | |
| 1. **Current headache** | | **Yes** | **No** |
| 1. Time of first attack | | | |
| Within 1 h after stroke onset | | 1 | 0 |
| 1-24 h after stroke onset | | 1 | 0 |
| 24 h later after stroke onset | | 1 | 0 |
| 1. Frequency | | | |
| ≤2 days every month | | 1 | 0 |
| 2-7 days every month | | 1 | 0 |
| 7-14 days every month | | 1 | 0 |
| >15 days every month | | 1 | 0 |
| 1. Duration | | | |
| ≤30 minutes | | 1 | 0 |
| 30 min- 4 h | | 1 | 0 |
| 4 h- 24h | | 1 | 0 |
| >1 day | | 1 | 0 |
| 1. headaches occur on any particular day of the week or time of day | | 1 | 0 |
| Specify: | | | |
| 1. Severity (0-10, “0” means no pain, “10” means unbearable pain) | | | |
| Mild (0-3) | | 1 | 0 |
| Moderate (4-6) | | 1 | 0 |
| Severe (7-10) | | 1 | 0 |
| 1. Headache types: | | | |
| Tension-type like | | 1 | 0 |
| Migraine like | | 1 | 0 |
| Other: | | | |
| 1. Headache characteristics: | | | |
| Pulsating | | 1 | 0 |
| Pressing | | 1 | 0 |
| Stabbing | | 1 | 0 |
| Other: | | | |
| 1. Headache location: | | | |
| Left side | | 1 | 0 |
| Right side | | 1 | 0 |
| Both | | 1 | 0 |
| 1. Aura symptoms before headache | | | |
| Group A | Blurred vision | 1 | 0 |
|  | Dark spots/floaters | 1 | 0 |
|  | Bright light/glare | 1 | 0 |
|  | Distorted vision | 1 | 0 |
|  | Other: | | |
| Group B | Nausea | 1 | 0 |
|  | Vomiting | 1 | 0 |
|  | Light sensitivity | 1 | 0 |
|  | Noise sensitivity | 1 | 0 |
|  | Numbness | 1 | 0 |
|  | Weakness | 1 | 0 |
|  | dizziness | 1 | 0 |
|  | Fever | 1 | 0 |
|  | Others: | | |
| Group C | Difficulty speaking | 1 | 0 |
|  | Enunciation unclear | 1 | 0 |
|  | Other: | | |
| Group D | Limb weakness | 1 | 0 |
|  | Ataxia | 1 | 0 |
|  | Other: | | |
| Other: | | | |
| 1. Accompanied symptoms | | | |
| Nausea | | 1 | 0 |
| Vomiting | | 1 | 0 |
| Neck pain | | 1 | 0 |
| Numbness | | 1 | 0 |
| Weakness | | 1 | 0 |
| Difficulty speaking | | 1 | 0 |
| Tearing | | 1 | 0 |
| Dizziness | | 1 | 0 |
| Other: | | | |
| 1. Aggravation by: | | | |
| Exercise | | 1 | 0 |
| Stress | | 1 | 0 |
| Change in weather | | 1 | 0 |
| Alcohol | | 1 | 0 |
| Smoke | | 1 | 0 |
| Noise | | 1 | 0 |
| Other: | | | |
| 1. **Headache impact test (HIT-6)** | | | |
| 1. How often does severe pain occur when you have a headache   □Never □seldom □sometimes □often □always | | | |
| 1. Whether headaches often limit your ability to perform daily activities, such as housework, work, school or social activities   □Never □seldom □sometimes □often □always | | | |
| 1. How often do you wish you could lie down and rest when you have a headache   □Never □seldom □sometimes □often □always | | | |
| 1. In the last 3 months, have you felt tired frequently due to headaches and felt unable to perform well during work days or daily activities   □Never □seldom □sometimes □often □always | | | |
| 1. In the last 3 months, have you often felt bored or upset due to headaches   □Never □seldom □sometimes □often □always | | | |
| 1. In the last 3 months, have you often had headaches that prevented you from focusing on work or daily activities   □Never □seldom □sometimes □often □always | | | |
| Never-6 seldom-8 sometimes-10 often-11 always-13 | | | |
| Total: | | | |
| 1. Treatment for headaches: | | | |
| Medicines | | 1 | 0 |
| Acupuncture | | 1 | 0 |
| Chiropractic | | 1 | 0 |
| Physical therapy | | 1 | 0 |
| Other: | | | |
| Specify: (types, usage and dosage):  1.  2.  3.  4. | | | |

**TableS2 Univariate and stepwise multiple regression analysis of risk factors for PHPIS**

|  | **Univariate analysis** | | **Multiple analysis** | |
| --- | --- | --- | --- | --- |
|  | **OR (95% CI)** | ***P-*value** | **OR (95% CI)** | ***P-*value** |
| **Age** | 0.975(0.949-1.002) | .065 | 0.968(0.939-0.997) | .033^a^ |
| **Females** | 1.788(0.999-3.200) | .050 | 2.395(1.269-4.520) | .007^a^ |
| **Hypertension** | 0.698(0.389-1.251) | .227 | 0.578(0.305-1.095) | .092 |
| **Diabetes** | 1.010(0.543-1.878) | .975 |  |  |
| **Hyperlipemia** | 0.762(0.289-2.006) | .582 |  |  |
| **Atrial fibrillation** | 0.627(0.144-2.735) | .534 |  |  |
| **Previous stroke** | 1.544(0.771- 3.093) | .220 | 2.105(0.984-4.502) | .055 |
| **Smoking** | 1.028(0.582-1.814) | .925 |  |  |
| **Alcohol** | 0.811(0.438-1.504) | .507 |  |  |
| **Initial NIHSS** | 0.967(0.900-1.039) | .357 | 0.928(0.852-1.011) | .086 |
| **ASPECT score** | 0.985(0.752-1.289) | .912 |  |  |
| **IVT** | 1.981(1.099-3.570) | .023 | 2.505(1.313-4.782) | .005^a^ |
| **Circulation** |  |  |  |  |
| Anterior | Ref. |  |  |  |
| Posterior | 2.605(1.357-5.003) | .004 | 2.188(1.110-4.311) | .024^a^ |
| Both | 1.970(0.936-4.144) | .074 | 2.085(0.962-4.519) | .063 |

Abbreviations: PHPIS, persistent headache attributed to past ischemic stroke; NIHSS, National Institute of Health stroke Scale; ASPECT, Alberta Stroke Program Early CT Score; IVT, intravenous thrombolysis; CI, confidence interval; OR, odds ratio.

Binary logistic regression, backward, adjusted analysis includes observed categorical variables (sex, hypertension, hyperlipemia, diabetes, atrial fibrillation, previous stroke, smoking, alcohol intake, IVT and circulation) and continuous variables (age, NIHSS and ASPECT scores).

^a^ *p* < .05 vs. non-IVT group
